# Synthetic indicators to analyze work-related physical and psychosocial risk factors: evidence from the European Working Conditions Survey

**DOI:** 10.1007/s11135-023-01617-8

**Published:** 2023-02-21

**Authors:** Stefania Capecchi, Carmela Cappelli, Maurizio Curtarelli, Francesca Di Iorio

**Affiliations:** 1 Department of Political Sciences, University of Naples Federico II, Naples, Italy; 2 Department of Humanities, University of Naples Federico II, Naples, Italy; 3 Prevention and Research Unit, European Agency for Safety and Health at Work, Bilbao, Spain

**Keywords:** Self-assessed health, Well-being, Psychosocial risks, Physical risks, Working conditions

## Abstract

In modern workplaces, alongside physical, chemical, and biological hazards, other risks are linked to the organisation of work and to the nature of the work itself. This paper investigates the association between workers’ well-being and both psychosocial and physical risk factors at work proposing a synthetic measure suitable to generate insights on well-being at work and on individual risk factors. Exploiting data from the European Working Conditions Survey, we select as response variable the “self-assessed health”. As this proxy of well-being is measured on a Likert scale, Ordered Probit analyses are run, and respondents’ profiles are illustrated. Then, a Principal Component Analysis is carried out to build two synthetic measures summarising the selected risk determinants. The resulting first principal components are subsequently used as synthetic indicators in further, simplified, Ordered Probit models to explain the impact of different sets of risks on perceived health. Such a methodology allows for a straightforward interpretation of the results since many different risk drivers are replaced by two continuous synthetic indicators. Our findings, in line with existing research, confirm that both types of risk factors do exert a substantial impact on workers’ health, although the psychosocial determinants seem to be more prominent.

## Introduction

This paper aims to analyse the association between workers’ well-being with psychosocial and physical risk factors in the workplace, identifying if—and how much—well-being is differently impacted by each of the two set of risk factors. The main purpose of our study is to build a synthetic measure suitable to generate insights on well-being at work and on individual risk factors.

Using the conceptual framework by the European Union Agency for Occupational Safety and Health (EU-OSHA 2013), we analyse the sixth European Working Conditions Survey (EWCS) data to detect how and to what extent selected determinants may influence subjective well-being, measured as a proxy by the self-assessed health (SAH).

Studies on such topics frequently employ the ratings given by respondents to each item in a Generalized Linear Models (GLM) framework (Agresti 2010). In survey questionnaires as that of the EWCS the items are often numerous, thereby making the identification of relevant determinants challenging. In our study, a selection (based on existing research) of reliable information related to physical and psychosocial risks in the workplace was carried out prior to the analysis, also considering that some of the variables affecting workers’ well-being can overlap, presumably because they measure very similar concepts. Their impacts may therefore become difficult to detect, especially considering that the GLM involve a large number of variables, which are potentially correlated to each other. This circumstance affect the interpretation of the results and may prevent general readership and decision makers to appreciate the role of specific determinants, thus making it difficult to acknowledge any risk assessment and management practices in the workplace.

To deal with this issue, in this paper we employed a Principal Component Analysis (PCA), a strategy to deal with a considerable number of variables and that may sometimes lead to overcome the composite indicators (see, among others, Nardo et al. 2005 and OECD and JRC 2008). The PCA is here carried out to calculate two distinct synthetic indicators, for the physical (*PC1.Phy*) and the psychosocial (*PC1.PS*) determinants, respectively. These indicators are then used in a simplified GLM model to analyse their impact on workers’ well-being. In such a way, the contribution of each risk determinant can be more easily evaluated by means of its factor loading in the construction of the PCA.

Our findings display that both categories of risk factors do have a significant impact on workers’ well-being measured by the SAH, although psychosocial risk factors seem to exert a greater effect. Attempts of measuring the interaction between physical and psychosocial risk factors at work and their impact on workers’ well-being, especially using synthetic indicators, appear to be limited in existing research. Thus, our study aims to contribute to fill this research gap.

The paper is organized as follows. Section 2 presents a brief overview of the literature on well-being and working conditions. Section 3 depicts the data employed, the identification of risks’ determinants in the workplace, and the distributions of the individual responses. Section 4 details the proposed empirical strategy to model responses and to build the two synthetic indicators of risk at work. Section 5 describes the implemented models, the proposed synthetic indicators, and discusses the empirical findings. In Sect. 6 some concluding remarks are provided also with respect to policy implications.

## Well-being at work as a multifaceted concept

Subjective well-being is a multidimensional concept encompassing several related phenomena, including emotional responses, feelings, and overall judgements of life satisfaction and its multifaceted domains, such as family life, job, health, and so on (WHO 2012; Bryson et al. 2014). In line with the initiatives, which existed at the global level since the 1990s, aimed at assessing social and economic progress through measures that go beyond the classical macroeconomic indicators (World Bank 1990), a sizeable literature, also inspired by the seminal work of Stiglitz et al. (2009), has been published in the wake of the “Beyond GDP movement” (see, among others, OECD 2013; Durand 2015; Bacchini et al. 2021). A branch of the literature has mainly investigated the determinants of life satisfaction, as a proxy of the overall well-being, at the individual-level whereas others aim at examining country-specific determinants of life satisfaction (Bjørnskov et al. 2008; Pittau et al. 2010). Furthermore, three main aspects can be classically distinguished within the concept of subjective well-being, as pointed out by Steptoe et al. (2012): evaluative well-being, affective or hedonic well-being, and eudemonic well-being.

More in general, the guidelines on the measurement of well-being developed by the OECD (2013) comprise “all of the various evaluations, positive and negative, that people make of their lives, and the affective reactions of people to their experiences” (OECD 2013, p. 29). Examples of life circumstances include health, education, work, social relationships, built and natural environments, security, civic engagement and governance, housing, and work-life balance (WHO 2012).

When it comes to health issues in particular, it can be essential to build an articulated indicator system. Health is indeed considered one of the main aspects which matters to well-being and evidence displays that a good health is correlated with higher life satisfaction (British Office for National Statistics 2011). As widely recognised in the literature over the last two decades, the relationship between health and well-being is indisputably complex and it is not just one-way: health influences well-being and well-being itself influences health (Howell et al. 2007; Pressman and Cohen 2005; Stoll et al. 2012; Steptoe et al. 2012; WHO 2012). There is also evidence about correlations between well-being and physical health outcomes such as cardiovascular health, improved immune system response, higher pain tolerance, reproductive health, lower pain, greater pain tolerance, and increased longevity (United Kingdom Department of Health 2014). On the other hand, health is reported to influence well-being through both its physical and mental health components, considering their strong interconnections. Being associated with numerous benefits related to individuals’ health, family and economic circumstances, well-being is an outcome meaningful to the public and relevant in the area of public health (Diener et al. 2009).

In Psychology, Howell et al. (2007) used the term *well-being* for positive psychological constructs that are measured (e.g., positive affect, optimism) or manipulated (in a positive emotion induction), contrasted by the term *ill-being* for negative psychological constructs (e.g. negative moods, stress, depression).

In light of the intricate network of interactions underlying social measurement processes in complex societies, a proposal by Arcagni et al. (2021) has raised new critical considerations in order to increase awareness of the inherent limitations of current practices used to measure social issues and in particular to effectively evaluate policies directed towards well-being and social sustainability. As an instance, other scholars (Bacchini et al. 2020, among others) have highlighted several limits in the use of the composite indicators for comparison along the time.

### Well-being at work

Work has long been recognised as having important (both positive and negative) impact on health and well-being, as effectively summarized by Litchfield et al. (2016). In modern workplaces -alongside to physical, chemical and biological risks, depending mostly on the type of industry- hazards are frequently related more to work organization and the nature of work itself (EU-OSHA 2013), rather than to specific agents, harm is therefore more psychological than physical. In fact, the work factors that can affect psychological (but also physical) health usually refer to job content, work organisation and management, their environmental and organisational conditions, as well as to worker’s competencies and needs. This interaction can prove to be hazardous to employee’s health through their perceptions and experience (EU-OSHA 2013).

The literature provides a comprehensive account of the job characteristics whose absence or poor-quality result in psychosocial risk factors that can affect workers’ health and well-being (Bryson et al. 2014; Brill 2021). These aspects include job content (i.e.: variety of tasks, correct matching of skills and tasks), job demands (i.e.: workload, work intensity, deadlines), control (i.e.: control over workload, participation in decision-making), work schedule, work-life balance, role in organisation, relationships at work (with colleagues and managers), physical environment and equipment (including work equipment availability, suitability and maintenance, environmental conditions such as light, noise, etc.), career development and prospects, and organisational culture and function (i.e.: communication, problem solving, definition of organisational objectives). It can be observed that when such characteristics result in psychosocial risks, they more frequently impact on workers’ health (and well-being) through a stress-mediated pathway. Within the field of occupational health and safety, the well-established “hazard-harm pathway model” to interpreting the association between exposure to occupational hazards and employee’s safety and health, was adapted to include and account for psychosocial risks (EU-OSHA 2013, p. 3).

Well-being at work has been extensively analysed within research focusing on job quality and job characteristics (among others: Hackman and Oldham 1976). A substantial part of the literature originates from the pivotal contribution of Karasek (1979) who conceptualised the “Job Demand-Control” (JD-C) model, according to which jobs involving high demands (resulting from intensification of work, increased workload) and low control (limited freedom to make decisions about how to organise and carry out own work) are related to higher levels of occupational stress. The “Job Design-Resource” (JD-R) model, conceptualised by Demerouti et al. (2001), considers that organisational job factors necessarily interact with job design. Demands are defined as those elements that entail physical or psychological effort; whereas resources, e.g., job characteristics, enable the worker to perform the required tasks. As underlined by Bakker and Demerouti (2014, p. 38): “Whereas job design theories have often ignored the role of job stressors or demands, job stress models have largely ignored the motivating potential of job resources. JD-R theory combines the two research traditions, and explains how job demands and resources have unique and multiplicative effects on job stress and motivation”. For a review of early and contemporary research on job design theory in organizations, see Oldham and Fried (2016).

Other scholars highlight that there is a considerable amount of evidence indicating that a positive correlation between well-being and an employee’s job performance does exist (Askitas and Zimmermann 2015; Bryson et al. 2014; Guzi and de Pedraza 2015, among others): results even display that higher level of worker’s well-being can lead to higher levels of job performance. The debate on these topics is part of a much broader body of research on job quality (Osterman 2013, Cazes et al. 2015; Padrosa 2021) and its relationship with health, both physical and psychological (see, for example, Henseke 2018).

It is worth mentioning that subjective characteristics (such as gender, age, personality, genes, education, and ability) also exert an impact on well-being in a number of ways. On the one hand, depending on their specific characteristics, the individuals can have a different perception or awareness of psychosocial risks and be resilient, with different outcomes in terms of well-being and perceived health conditions. On the other hand, individual attributes are inextricably linked to job and workplace factors and therefore are related to different levels of well-being (EU-OSHA 2017): for example, educated workers get access to better jobs; jobs held by older employees may offer more autonomy and higher income; immigrants work frequently in bad jobs (Bryson et al. 2014; Stoll et al. 2012).

Because it is subjective, well-being is usually measured with self-assessments. Self-reported well-being can be assessed in different ways, depending on whether well-being needs to be measured as a clinical outcome, a population health outcome, for cost-effectiveness studies, or for other purposes (Centers for Disease Control and Prevention 2018; WHO 2012). Some studies support the use of single items (e.g., global life satisfaction) to measure well-being parsimoniously. More specifically, and with all the caveats due to possible bias associated with the self-assessment, also in terms of comparability of results over space and time, well-being in the workplace is often assessed by proxies, as the level of overall job satisfaction or for some specific job features (Bryson et al. 2014; Maxwell 2015).

## Data

Data employed for this study stem from sixth wave of the European Working Conditions Survey (EWCS) carried out in 2015 by the European Foundation for the Improvement of Living and Working Condition.^1^ A sample of 43,850 employees and self-employed workers in 35 European countries, representative of the employed population, were interviewed: the European Union (EU) Member States (28 countries in 2015) plus Norway, Switzerland, Albania, the Former Yugoslav Republic of Macedonia, Montenegro, Serbia, and Turkey. The survey questionnaire is extremely rich and covers a wide number of topics referred to worker characteristics, working conditions, work environment factors, impacts on worker’s health (Eurofound 2017), thus representing a prominent data source^2^ for comparison across countries, occupations, sectors, and age groups. The employed data are the most up-to-date statistically representative information. The 2020 round of the EWCS was abruptly interrupted at the beginning of the fieldwork^3^ due to the outbreak of COVID-19. To control for extreme heterogeneity within the data, our study considers only the 2015 EU Member States.

As previously mentioned, several studies relate higher subjective worker’s well-being with higher self-assessed health (SAH), and studies based on longitudinal data also show a strong effect of health on subjective well-being (Dolan et al. 2006). Therefore, given its relationship with well-being and considering the limitations of other indicators such as those measuring job satisfaction, in this paper we adopt SAH as a parsimonious measure of workers’ well-being, using question Q75—“How is your health in general? Would you say it is: 1 Very good; 2 Good; 3 Fair; 4 Bad; 5 Very bad”, measured on a 5-point scale.

Common individual characteristics here considered are *gender* (question Q2a, expressed by a dummy variable where female = 2); *age* (in years, Q2b); education level (from the original Q106) is defined as a dummy where university degree = 1 (*tertiary*). Given the high number of missing values with reference to income, to investigate the effect of the individual’s economic status, information is derived from Q100: “Thinking of your household’s total monthly income, is your household able to make ends meet” (*make-ends-meet*) rated on a six-point wording scale from “Very easily” (1) to “With great difficulty” (6). Job features described by answers to Q2d and Q11, are represented by two dummies: full-time vs. part-time job (*fulltime*, where full-time = 1) and permanent vs. non-permanent job (*permjob*, where permanent job = 1). Moreover, we reckon the number of working days per week (Q26, *d4w*) and of hours weekly spent at work (Q24, *whours*).

We consider two sets of drivers for SAH: *physical risk factors* and* psychosocial risk factors* at work. Table 1 lists the questions of interest from which the variables under study originate. The question corresponding codes, as in the original dataset, and the variable labels as they are used in the figures, are also provided in Table 1. For each question, we also report the scales as used in the elaborations oriented from the lowest to the highest level of agreement.

**Table 1:** List of selected questions and related variables (physical and psychosocial risk factors)

Variable label	Original question code	Question	
*Physical risk factors*			
Q29. Please tell me, using the following scale, are you exposed at work to…? (1 = never; 7 = always)			
Vibra	Q2A	Vibrations from hand tools, machinery, etc	
Noise	Q29B	Noise so loud that you would have to raise your voice to talk to people	
Htemp	Q29C	High temperatures which make you perspire even when not working	
Ltemp	Q29D	Low temperatures whether indoors or outdoors	
Smoke	Q29E	Breathing in smoke, fumes (such as welding or exhaust fumes), powder or dust (such as wood dust or mineral dust)	
Vaps	Q29F	Breathing in vapours such as solvents and thinners	
Chem	Q29G	Handling or being in skin contact with chemical products or substances	
Tobs	Q29H	Tobacco smoke from other people	
Infect	Q29I	Handling or being in direct contact with materials which can be infectious, such as waste, bodily fluids, laboratory materials, etc	
Q30. Please tell me, using the same scale, does your main paid job involve…? (1 = never; 7 = always)			
Painp	Q3A	Tiring or painful positions	
Liftp	Q30B	Lifting or moving people	
Loads	Q30C	Carrying or moving heavy loads	
Sitting	Q30D	Sitting	
Repet	Q30E	Repetitive hand or arm movements	
Comput	Q30I	Working with computers, laptops, smartphones etc	
Costum	Q30F	Dealing directly with people who are not employees at your workplace such as customers, passengers, pupils, patients, etc	
Angry	Q30G	Handling angry clients, customers, patients, pupils etc	
Emod	Q30H	Being in situations that are emotionally disturbing for you	
*Psychosocial risk factors*			
Fitwell	Q44	In general, how do your working hours fit in with your family or social commitments outside work? (1 = not at all—4 = very well)	
Q45 How often in the last 12 months, have you…? (1 = never—5 = always)			
Worry	Q45A	kept worrying about work when you were not working	
Tired	Q45B	felt too tired after work to do some of the household jobs which need to be done	
TimeIwant	Q45 C	found that your job prevented you from giving the time you wanted to your family	
Q49. And, does your job involve (1 = never – 7 = always)			
Hspeed	Q49A	working at very high speed	
Deadl	Q48B	working to tight deadlines	
Q61. For each of the following statements, please select the response which best describes your work situation. (1 = never—5 = always)			
SupportC	Q61A	Your colleagues help and support you	
SupportM	Q61B	Your manager helps and supports you	
Improve	Q61D	You are involved in improving the work organisation or work processes of your department or organisation	
Hsay	Q61E -	You have a say in the choice of your work colleagues	
Tbreak	Q61F	You can take a break when you wish	
Entime	Q61G	You have enough time to get the job done	
Welld	Q61H	Your job gives you the feeling of work well done	
Ideas	Q61I	You are able to apply your own ideas in your work	
Useful	Q61J	You have the feeling of doing useful	
Expected	Q61K	You know what is expected of you at work	
Fair	Q61L	You are treated fairly at your workplace	
Decisions	Q61N	You can influence decisions that are important for your work	
Hfeel	Q61O	Your job requires that you hide your feelings	
Q70. The next questions are about your workplace. To what extent do you agree or disagree with the following statements? (1 = never—5 = always)			
Apprec	Q70A	Employees are appreciated when they have done a good job	
Truste	Q70B	The management trusts the employees to do their work well	
Coop	Q70E	There is good cooperation between you and your colleagues	
Trustm	Q70F	In general, employees trust management	
Q89. To what extent do you agree or disagree with the following statements about your job? (1 = never—5 = always)			
Geton	Q89D	I generally get on well with my work colleagues	
Motiv	Q89E	The organisation I work for motivates me to give my best job performance	

*Source*: Authors’ elaboration on EWCS 2015 questionnaire.

Notice that, for most of the considered variables, we needed to conveniently reverse the original scales to make the wording of the questions consistent with the adopted scale for SAH.

Variable *dEU12* refers to the original 12 European Union Member States, those supposed to share similar political and socio-economic features.

Missing values and *“Don’t know*” responses have not been considered in the analyses; therefore, our sub-sample eventually consists of 19,996 individuals.

Women are 51% of the sample. 27.2% of respondents hold a university degree. About 81.2% of the sample have a permanent job; 78.6% have a full-time job and 67.4% work in the private sector. The average age is 42 years (std error: 11.9). Respondents work on average 37 h per week (std. error: 10.5) on 5 working days.

**Fig. 1:**
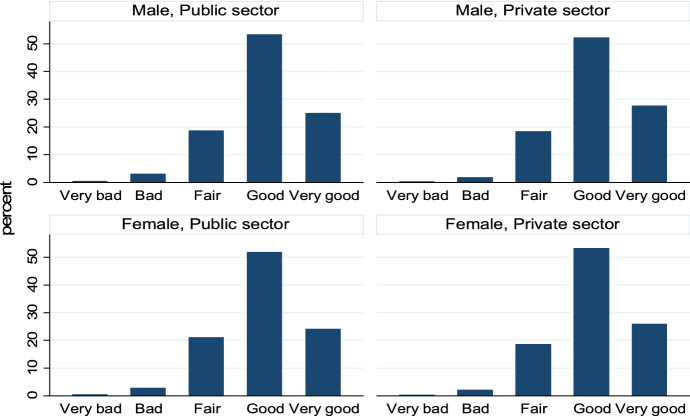
SAH frequency distribution by gender and sector. *Source*: Authors’ elaborations on EWCS 2015 data

The proportion of those claiming a bad or very bad SAH is 2.4%, while more than 77% report a positive or very positive evaluation (good: 52.6%; very good: 26%). As shown in Fig. 1, there are not prominent differences in the frequency distributions with regards to gender and sector. The descriptive statistics referring to all remaining variables are included in the Appendix (Tables 5, 6, 7).

## Empirical analysis

In surveys on topics referred to working population, such as well-being and self-reported working conditions are usually measured by means of scales administered to respondents, who are asked to select a response category out of a list. This is the case of the variable SAH chosen for our analysis. Generally, in a modelling approach, the ordinal responses are used as they are when considered as explanatory variables. Consolidated statistical procedures are those derived by GLM (Agresti 2010). Nevertheless, such classic model-based analysis, carried out employing this kind of explanatory variables, may be difficult to interpret by general readership, therefore composite and synthetic measures are largely preferred (OECD and JRC 2008). Several statistical approaches have been exploited in the field of well-being measurement (among others, Maggino 2016), even in a model-based perspective (see, e.g.: Capecchi and Simone 2019).

We propose to simplify the model-based analysis introducing synthetic measures obtained by Principal Component Analysis (PCA, e.g.: Jolliffe 2011).

The methodology implemented in this study involve several steps. First, given the nature of SAH, preliminary Ordered Probit analyses are run, based on explanatory drivers describing 15 physical and 26 psychosocial risk factors; 9 individual characteristics are also considered. Selected profiles are illustrated. A PCA is then carried out to obtain two distinct synthetic indicators summarising the selected sets of risk drivers. The resulting synthetic indicators – the first principal components (PCs) for each set of risks—are subsequently used as explanatory variables in further, simplified, Ordered Probit models. This strategy allows to make the interpretation of the results more straightforward, since 42 different drivers for physical and psychosocial risk at work are replaced by two continuous synthetic indicators: PC1.Phy and PC1.PS, respectively.

## Models and findings

In Orderd Probit models an underlying score is estimated as a linear function of the explanatory variables and a set of cutpoints. Even though the explanatory variables are ordinal, consistent support has been found for treating them as approximately continuous (Norman 2010; Johnson and Creech 1983), and as proxies of non-observable latent variables (Agresti 2010). In this framework, the probability of observing the outcome *j* corresponds to the probability that the estimated linear function, plus random error, is within the range of the cutpoints estimated for the outcome. The model has the following expression:

1 $${\text{Pr}}\left( {{\text{Y}}_{{\text{i}}} = {\text{ j}}} \right) = {\text{Pr}}({\text{k}}_{{{\text{j}} - {1}}} < \upbeta_{{1}} {\text{x}}_{{{\text{1i}}}} + \upbeta_{{2}} {\text{x}}_{{{\text{2i}}}} + \, . \, . \, . + \, \upbeta_{{\text{h}}} {\text{x}}_{{{\text{hi}}}} + {\text{ u}}_{{\text{i}}} \le {\text{ k}}_{{\text{j}}} )$$

where u_i_ ∼ N (0, σ^2^), β_1_... β_h_ (the coefficients associated to *h* drivers) and cutpoints k_1_... k_J−1_ are the parameters to be estimated, J is the number of possible outcomes, and i = 1...n refer to the individuals; finally, k_0_ is taken as − ∞ and k_J_ is taken as + ∞.

Estimates are obtained using STATA (version 14) where the dummy variables are treated as factors, σ^2^ = 1 and the constant term set to zero are the normalization constrains (see Veerbek 2004, Sect. 7.2.2). We estimate three models, all of them including the individual and job-related covariates. Model 1 considers as explanatory variables the physical risk factors only, Model 2 considers the psychosocial risks only, and Model 3 is a comprehensive one, allowing to verify how the two sets of risks interact with each other and the individual variables. Results are presented in Table 2.

**Table 2:** Ordered Probit estimates

SAH	Model 1 (physical risk factors)		Model 2 (psychosocial risk factors)		Model 3 (comprehensive)	
	Coef.	SE	Coef.	SE	Coef.	SE
Gender	− 0.083	0.018***	− 0.054	0.017**	− 0.083	0.018***
Age	− 0.029	0.001***	− 0.030	0.001***	− 0.030	0.001***
Tertiary education	0.112	0.020***	0.167	0.019***	0.136	0.020***
Permanent job	− 0.069	0.022***	− 0.056	0.022***	− 0.062	0.022***
Fulltime job	0.081	0.025***	0.057	0.025***	0.061	0.025***
Private Sector	0.054	0.018**	0.065	0.018	0.068	0.019**
Working hours	0.000	0.001	0.004	0.001***	0.005	0.001***
Working days per week	− 0.006	0.011	0.004	0.011	0.006	0.011
Make-ends-meet	0.133	0.007***	0.109	0.007***	0.096	0.007***
Vibrations	0.015	0.006**			0.009	0.007
Noise	− 0.031	0.006***			− 0.019	0.006***
High temperature	− 0.014	0.006***			− 0.006	0.006
Low temperature	− 0.014	0.007**			− 0.008	0.007
Smoke	− 0.011	0.007**			− 0.009	0.007
Vapours	− 0.010	0.009			− 0.007	0.009
Chemicals	− 0.016	0.007*			− 0.014	0.007**
Tobacco	− 0.018	0.008**			− 0.010	0.008
Infective materials	− 0.003	0.007			0.006	0.007
Painful positions	− 0.060	0.005***			− 0.043	0.005***
Lifting persons	0.008	0.007			0.021	0.008***
Loads	− 0.017	0.006***			− 0.007	0.006
Sitting	− 0.024	0.005***			− 0.015	0.005***
Repetitive movements	− 0.007	0.004**			− 0.013	0.004***
Computers	0.006	0.004***			0.010	0.004***
Emotional disturbing			− 0.028	0.005***	− 0.039	0.007***
Costumer			0.163	0.013***	0.020	0.004***
Angry clients			− 0.057	0.009**	− 0.011	0.006**
Fit well			− 0.014	0.005***	0.159	0.013***
Time I want			− 0.008	0.005***	− 0.044	0.009***
High speed			0.019	0.010**	0.001	0.006
Deadlines			0.027	0.009	0.002	0.005
Support. colleagues			0.004	0.007	0.023	0.010**
Support. manager			− 0.018	0.006***	0.024	0.009*
Improve work organization			0.005	0.006	0.001	0.007
Have a say			0.010	0.009***	− 0.016	0.007**
Take a break			0.048	0.012	− 0.001	0.006
Enough time			0.002	0.008	0.017	0.009*
Well done			0.009	0.011***	0.053	0.012***
Ideas			− 0.019	0.013	0.001	0.008
Useful			0.030	0.012	0.010	0.012
Expected			− 0.021	0.008	− 0.012	0.013
Fairly			0.012	0.006*	0.013	0.012
Decisions			0.008	0.010**	− 0.022	0.008***
Hiding feelings			− 0.005	0.012	0.016	0.006***
Appreciated			0.000	0.014	0.007	0.010
Trusted			0.049	0.010	0.000	0.012
Cooperation			0.067	0.014	0.000	0.014
Trusting manager			0.070	0.009***	0.043	0.010***
Get on well			0.109	0.017***	0.068	0.014***
Motivation			− 2.197	0.119***	0.063	0.009***
Dummy EU12	0.072	0.016***	− 1.278	0.111***	0.087	0.017***
cutpoint1	− 4.250	0.088	0.043	0.110	− 2.542	0.123
cutpoint2	− 3.355	0.077	1.671	0.110	− 1.614	0.115
cutpoint3	− 2.062	0.074	− 0.028	0.005	− 0.276	0.114
cutpoint4	− 0.476	0.073	0.163	0.013	1.368	0.114
Log-likelihood	− 20,451.04		− 20,025.27		− 19,875.58	
Likelihood ratio test						
(H_0_: model with no predictors)	3171.50***		4023.03***		4322.41***	
Pseudo-R^2^	0.0720		0.0913		0.0981	

***Significant at 1%; **significant at 5%; *significant at 10%

*Source*: Authors’ elaborations on EWCS 2015 data

In line with the existing literature, the individual characteristics and the main job characteristics are in general significant. Some of the risk variables lose their statistical significance when passing from the models for single risk set to a comprehensive Model 3, whereas a few ones do acquire significance. These results may originate from the circumstance that some questions can be considered “equivalent” by respondents and therefore lead to disperse information and artificially alter the variability in the sample. The dummy variable (*dEU12*), which identify the EU12 countries, is always significant, indicating that sharing similar socio-economic features does exert an impact on response pattern.

Overall, in the transition from the models for a specific risk set of risks to the comprehensive one, the psychosocial determinants of perceived health seem to have a more robust impact, as they undergo fewer variations in terms of statistical significance. This evidence may suggest that, in this dataset, the psychosocial risks do seem to exert a greater influence on workers’ perception of health as compared to the physical ones.

It is well known that for the intermediate response categories the interpretation of the coefficients in the Ordered Probit model is complex, since the coefficients’ sign and magnitude do not offer a clear indication of the partial effect extent for a given explanatory variable. Therefore, the impact of the covariates for such categories may result fundamentally ambiguous when observing the estimated coefficients (Daykin and Moffatt 2002; Greene 2008, Sect. 23.10). On the other hand, with respect to the extreme response categories on the scale (e.g. very good/very bad) the sign of the estimates can be meaningfully interpreted.

However, as the variables under consideration are numerous it may be more convenient to observe specific profiles of respondents to capture their effect. In fact, the effect on estimated probabilities of a drivers’ change depends on all model parameters, data, and dependent variable category of interest. The evaluation of such probabilities is based on specific values of the individual variables, hence defining some respondents’ profiles of interest.

Table 3 reports in the first two lines the observed frequencies for each SAH category in the whole sample, and the corresponding estimated probabilities obtained by using the coefficients of Model 3. Moreover, for each SAH category, the estimated probabilities for two types of respondents are reported, distinguishing by gender, type of contract (permanent, non-permanent), and age. In particular, age is considered at three different moments of working life: at 20, 40 and 65 years old.

**Table 3:** Estimated probabilities for selected respondents' profiles (Ordered Probit Model 3)

SAH		Very bad	Bad	Fair	Good	Very good
		*Overall observed frequencies*				
		0.003	0.022	0.190	0.526	0.260
		*Overall estimated probabilities*				
		0.001	0.011	0.17*0*	0.587	0.23*0*

Gender	Age	Private sector worker with tertiary education and full-time permanent job				
*Profile 1*						
Male	20	0.000	0.001	0.036	0.404	0.560
Female	20	0.000	0.001	0.042	0.429	0.528
Male	40	0.000	0.005	0.111	0.558	0.325
Female	40	0.000	0.007	0.126	0.570	0.297
Male	65	0.003	0.035	0.294	0.555	0.113
Female	65	0.004	0.041	0.316	0.540	0.099

Job type	Age	Private sector male worker with tertiary education and full-time job				
*Profile 2*						
Non-permanent	20	0.000	0.001	0.031	0.384	0.584
Permanent	20	0.000	0.001	0.036	0.404	0.560
Non-permanent	40	0.002	0.005	0.100	0.547	0.347
Permanent	40	0.003	0.005	0.111	0.558	0.325
Non-permanent	65	0.003	0.030	0.277	0.564	0.126
Permanent	65	0.003	0.035	0.294	0.555	0.113

*Source*: Authors’ elaborations on EWCS 2015 data.

The first profile refers to a worker with tertiary education, working in the private sector, with a full-time permanent job (distinguishing by gender and age). The second one considers a male worker, with tertiary education and a full-time job in the private sector (distinguishing by contract type and age). All the remaining determinants related to the risk factors are set to their mean value.

With respect to the Profile 1, it can be observed that the estimated probabilities for each SAH category (from “very bad” to “very good”) are quite similar for male and female respondents. The age of respondents is positively correlated to the SAH, that is young respondents are more likely to report good or very good health. The same can be said for Profile 2, where it can be observed that the contract type produces limited differences in the estimated probabilities, for each age group. Actually, temporary jobs are held by around 18% of the sample and are concentrated in the age range between 30 and 50 years old. At a disaggregated level, as the age varies, SAH decreases as expected, and the difference between workers in non-permanent jobs and workers in permanent jobs is still very limited and likely dependent on the small number of non-permanent workers over 50 in the sample (less than 1,000 in total in the considered sample).

### Derivation of the PCs as composite indicators

A PCA has been performed separately on the two sets of risk factors listed in Table 1. As the variables are ordinal, polychoric correlation has been considered (for details, see Olsson 1979). The main assumption is that ordered categorical variables arise polychotomising underlying continuous variables, then polychoric correlation can be regarded as an estimate of classical Pearson correlation coefficient and are read in the same way.

**Fig. 2:**
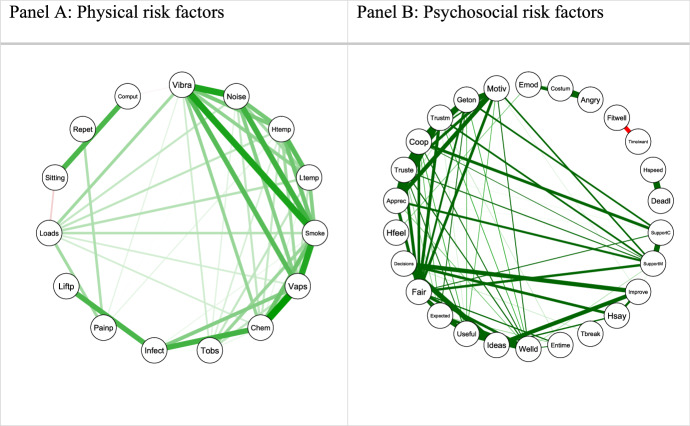
Polychoric correlations among risk factors (threshold 0.35 in absolute value). *Source*: Authors’ elaborations on EWCS 2015 data

Panel A in Fig. 2 displays the polychoric correlations among the physical risk factors*,* using a graph where each node represents a risk item, while lines connecting the nodes indicate the correlations among the items. The thicker the line the higher the correlation, while the colour denotes whether the correlation is positive (green) or negative (red). Stronger positive correlations can be noticed mainly between variables referring to vibrations (*vibra*), breathing in vapours (*vaps*), smoke and fumes (*smoke*), presence of chemical (*chem*) or infectious materials (infect), which represent physical risk factors present in the workplace (see Table 1). It can be noticed that the only negative correlation is that between two variables related to work-life balance (*fitwell* and *timeIwant*).

The eigenvalue associated with the first PC, say “PC1.Phy”, is 6.18, while the second largest is much smaller (1.59), thus PC1.Phy accounts alone for 41% (6.18/15, where 15 is the number of risk drivers) of the total variance suggesting that one dimension is able to adequately synthetize the data, summarizing the relevant information content, as also confirmed by the high significance of the test of the hypothesis that one component is sufficient.

**Fig. 3:**
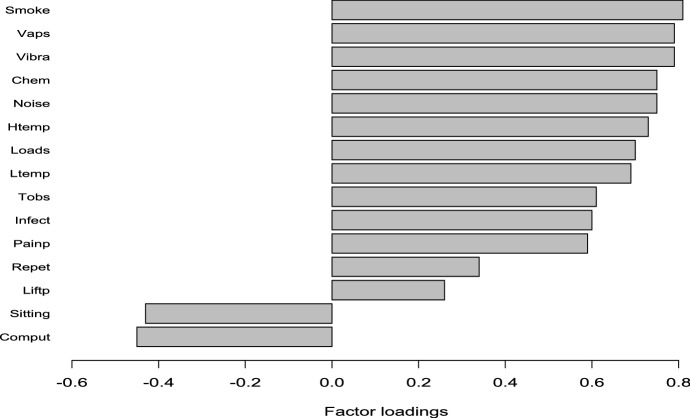
PC1.Phy factor loadings for the observed covariates. *Source*: Authors’ elaborations on EWCS 2015 data

An insight into the meaning of PC1.Phy is provided by the factor loadings reported in Fig. 3. Loadings are the coefficients of the linear combination of the original variables from which the PC is constructed. They are basically correlation coefficients between observed variables and the principal component; therefore, the loading magnitude measures the relevance of the risk driver. As usual rule of thumb, a variable should have a factor loading of at least 0.4 in absolute value. Items whose factor loadings are below 0.3 (or even below 0.4) provide little contribution and could be ignored.

Figure 3 shows that all covariates (but, consistently, *comput* and *sitting*) present a positive loading, thus, the higher the value of the covariates (which, given the direction of the scale employed for coding, indicates worse physical working conditions), the higher the value of the PC1.Phy indicator (the other way around for *comput* and *sitting*). Along PC1.Phy it is possible to identify, at one end, workers with a high exposure to physical risk agents (with positive high scores on PC1.Phy) and, at the opposite end, those in desk-based jobs (low negative score on the PC1.Phy). From the values in Fig. 3 we see that the variables more affecting the PC1.Phy are: *vibra*, *noise*, *htemp*, *smoke*, *vapours*, *chem* and *loads*. The only loadings below the 0.4 threshold are those of variables *repet* and *liftp*: nevertheless, in the subsequent analysis we keep them for sake of homogeneity with respect to the estimated Probit models above discussed.

**Fig. 4:**
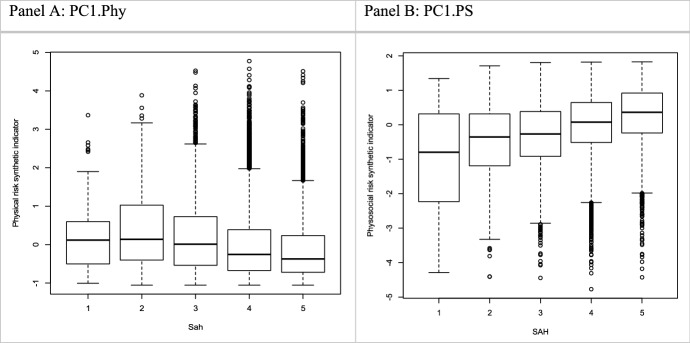
Distributions of the synthetic indicators versus the levels of SAH. *Source*: Authors’ elaborations on EWCS 2015 data

Since PC1.Phy ranges from better to worse physical conditions, it tends to be negatively correlated to SAH, as clearly displayed in Panel A of Fig. 4, which reports the boxplots of the distributions of the indicator against the levels of SAH. Despite the presence of several outliers, we can see that increasing levels of SAH (from “fair” to “very good”) are associated with lower levels of the indicator and, correspondingly, of the variables in the linear combination, specifically of those with the highest loading.

Similarly, an indicator has been derived for the set of variables describing the psychosocial risk factors. The corresponding polychoric correlations are plotted in Panel B of Fig. 2. Stronger positive correlations can be mainly observed between the factors referring to the relationships with colleagues and manager, the emotional sphere, such as feeling useful and fairly treated, being supported, trusted, involved in work organization, and being appreciated.

**Fig. 5:**
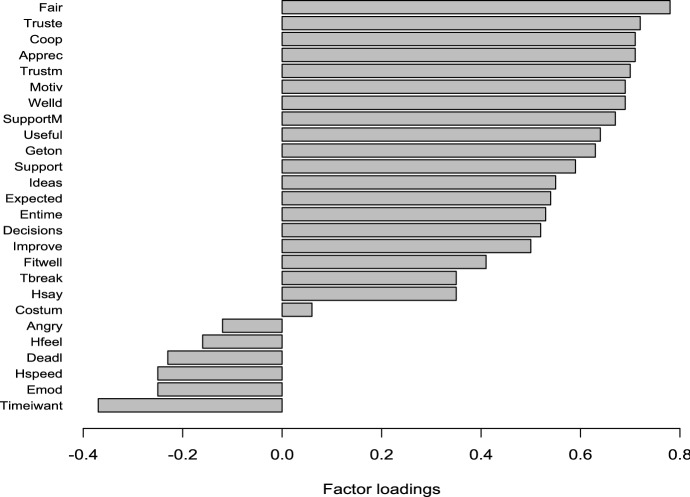
PC1.PS factor loadings for the observed covariates. *Source*: Authors’ elaborations on EWCS 2015 data

In this case, the eigenvalue associated to the first PC (let “PC1.PS”) is 7.34, while the second largest is 2.85. As expected, PC1.PS explains a lower percentage of the total variance (28%) as the number of items in this case is much larger. Nevertheless, one dimension (PC1.PS) captures most of the information in the data and, also in this case, the hypothesis test that one component is sufficient is highly significant. Looking at the factor loadings reported in Fig. 5, it can be noted that most of the variables are positively correlated to PC1.PS and show a loading which is well greater than the 0.4 threshold.

Particularly, in this case, PC1.PS is mainly explained by the variables *fair, apprec, coop, truste, trustm, motiv, welld, supportM, geton* and *useful.* The higher the value of these determinants, which denote a positive and motivating work environment, the higher the value of PC1.PS. In this case, higher values of the indicator denote healthier working conditions, thus PC1.PS is positively correlated to SAH, as shown in Panel B of Fig. 4 where the distribution of the PC1.PS is plotted against the levels of SAH. Despite the presence of outliers, clearly, increasing levels of SAH correspond to higher levels of the synthetic indicator and of the above-mentioned variables which provide the highest contributions to the variance of the corresponding linear combination.

### Ordered probit models including synthetic indicators

Based on the findings of the PCA, three further Ordinal Probit models have been estimated considering the respondents’ characteristics and using the synthetic indicators of risks. As reported in Table 4, Model 4 and Model 5 consider the impact on SAH of PC1.Phy and PC1.PS, respectively, whereas Model 6 simultaneously considers both the indicators. In all cases, the two indicators, either together or alone, turn out to be significant, confirming that they may provide an effective synthesis of the underlying variables which do exert some impact on SAH.

**Table 4:** Ordered probit with synthetic indicators for *physical* and *psychosocial risk factors*

SAH	Model 4 (PC1.Phy)		Model 5 (PC1.PS)		Model 6 (comprehensive)	
	Coef	SE	Coef	SE	Coef	SE
Gender	− 0.108	0.017***	− 0.032	0.017*	− 0.088	0.017***
Age	− 0.029	0.001***	− 0.030	0.001***	− 0.030	0.001***
Tertiary education	0.094	0.019***	0.148	0.019***	0.096	0.019***
Permanent job	− 0.074	0.021***	− 0.073	0.021***	− 0.082	0.022***
Fulltime job	0.077	0.025***	0.065	0.025**	0.060	0.025***
Private Sector	0.039	0.018	0.051	0.018**	0.051	0.018**
Working hours	− 0.001	0.001	0.000	0.001**	0.001	0.001
Working days per week	− 0.008	0.011	− 0.002	0.011	− 0.002	0.011
Make ends meet	0.139	0.007***	0.114	0.007***	0.103	0.007***
PC1.Phy	− 0.190	0.010***			− 0.148	0.010***
PC1.PS			0.281	0.009***	0.261	0.009***
dEU12	0.070	0.016***	0.096	0.016***	0.092	0.016***
cutpoint1	− 3.789	0.082	− 3.829	0.083	− 3.949	0.083
cutpoint2	− 2.904	0.070	− 2.924	0.070	− 3.037	0.071
cutpoint3	− 1.621	0.068	− 1.619	0.068	− 1.720	0.068
cutpoint4	− 0.046	0.067	− 0.013	0.067	− 0.103	0.067
Log-likelihood	− 20,562.26			− 20,255.55	− 20,145.54	
Likelihood ratio test (H_0_: model with no predictors)	2949.05***		3562.47***		3782.48***	
Pseudo-R^2^	0.0669		0.0808		0.0858	

*Source*: Authors’ elaborations on EWCS 2015 data

In particular, the negative effect of the PC1.Phy indicator is due to the fact that the underpinning items represent detrimental physical working conditions. Hence, they are negatively correlated to SAH: an increasing exposure to such risk factors (higher values of the employed scale and, correspondingly, of the PC1.Phy indicator) is associated with worse SAH levels. This is especially true for those items having a strong effect on the PC1.Phy indicator (see Fig. 3). Conversely, the positive effect of the PC1.PS indicator on SAH is due to the positive correlations to SAH of most of the items whose corresponding loadings are mainly positive (see Fig. 5). In fact, many of the items summarized by the PC1.PS denote a constructive and motivating working environment.

In these simplified Ordered Probit models, the dummy variable *dEU12* turns out to be always significant. Also, respondents’ characteristics remain significant in the same way, in all the models.

Concerning the physical risk factors, when comparing results from Table 2, it can be noted that although the variables *vaps* (breathing vapours) and *infect* (referred to the exposure to biological hazards) are not significant in Model 1, they are included in the synthetic indicator PC1.Phy with a loading close to *vibra* and *noise* (0.79 respectively, see Fig. 4). On the contrary, the variable *liftp*, which is significant in Model 1, presents small loadings (0.26) to the variance of PC1.Phy. However, there is quite a broad agreement between the significance of drivers in Model 1 and the content of the synthesis provided by PC1.Phy. When, on the other hand, we focus on psychosocial risk factors in Model 2, results display that a number of variables which are related to the workplace social environment and to the relationships with colleagues and manager (*supportc*, *supportm*, *useful*, *expected*, *fair*, *truste*, *trustm*, *apprec*, *coop*) are either not significant or weakly significant. Nevertheless, these variables present high loadings for PC1.PS, ranging from 0.54 to 0.71. Thus, for the psychosocial risk covariates, we may conclude that the first PC provides a synthesis of the information that is somewhat complementary with respect to the standard model with the original covariates, as it disentangles a specific cluster of psychosocial risk factors affecting SAH.

## Conclusions

The objective of this paper was to explore the association of workers’ well-being -measured by self-assessed health (SAH)- with *psychosocial* and *physical* risk factors in the workplace, introducing synthetic measures suitable to identify if -and how much- the two sets of risk factors exert an impact on worker’s well-being.

For our purposes we used data from the sixth EWCS and carried out Ordered Probit models to measure the effect of specific items, operationalising the physical and psychosocial risks. The estimated standard Ordered Probit models confirmed that both types of risk factors have a significant effect on SAH.

Some of the *psychosocial risk factors* are not significant in any of the models (or are weakly significant), while other variables display a strong correlation with SAH. This is the case of variables related to work-life balance (*fitwell* and *timeIwant*) or to a positive and motivating work environment, in which workers have a sense of fulfilment with work (*welld*), are motivated (*motiv*), are consulted and participate in decisions (*hasay, decisions*), are supported by management and trust managers (*supportm*, *trustm*) and have good relationships with colleagues (*geton*). Our findings are in line with existing literature, although they seem to point to the fact that, among the aspects most frequently indicated in the literature as related to well-being, only few of them– and in particular those related to the social environment at work and the work-life balance– are those contributing the most to worker’s well-being. Therefore, those aspects not *intrinsically* related to work seems to be the most impacting on workers well-being.

Among the *physical risk factors*, those related to vibrations, high or low temperatures, and tobacco smoke significantly impact on SAH in the model including only this type of risk factors, but cease to be significant in the comprehensive model. On the other hand, all the physical risk factors related to positions or movements during work (*painp*, *liftp*, *loads*, *sitting*, *repet*, *comput*) display a significant correlation with SAH. Such results are in line with literature that indicates that the worker’s physical environment and the equipment (including work equipment availability, suitability and maintenance, environmental conditions such as light, noise, etc.) also have an impact on well-being, although in this case risk factors directly related to physical efforts appear to be the most relevant, considering the strong association with the well-being as expressed by the SAH variable.

When considering the two types of risk factors together, it seems nevertheless that the psychosocial risks do exert a greater influence on workers’ perception of health as compared to the physical ones.

The introduction of two synthetic indicators, implemented using a Principal Component Analysis (PCA) for each of the two sets of risk factors, makes the interpretation more straightforward. Such indicators are the first Principal Component computed for each set (PC1.Phy for physical risk factors, and PC1.PS combining all the psychosocial drivers), accounting for most of the variance providing an effective synthesis of the information. These measures have subsequently been included in further Ordered Probit models for SAH, and they turned out to be statistically significant. Such evidence may suggest that the provided synthesis is to some extent complementary to the standard models.

The added value of building synthetic measures relies on that they allow either for simplifying a model-based analysis or for disentangling specific drivers of work-related well-being, as long as they are actually carriers of information, with the additional advantage of removing redundancies and obtaining more “robust” models.

In conclusion, the results presented allow to respond to our research questions in terms of strength and type of risk factors which seem to impact more on worker’s health and well-being in European workplaces.

It is important to also stress the limitations of this exercise, which stem directly from the data used and the survey tool itself. First and foremost, the physical risk factors are not extensively surveyed in the case of the EWCS and therefore refer only to a subset of respondents that is too limited to obtain reliable estimates. This is due to several reasons, such as: the specificities of this type of risk factors which cannot always be measured as self-reported; the circumstance that they exist more frequently in specific working contexts (manufacturing or construction) which employ only a reduced share of European workforce; the fact that European and national legislations have targeted this type of risk factors for several decades, now resulting in their steadily decrease. Thus, ad hoc surveys investigating physical risk factors where they mostly exist would be the right solution to provide more reliable analysis.

Furthermore, empirical evidence suggests that similar or highly correlated questions may be perceived as repetitive as confirmed by the circumstance that only the first principal component in the two groups of selected variables is significant. In fact, there is no clear division between the loadings that allow to characterize the main components, no great contrasts are captured, and there is a major noise. All in all, a questionnaire including fewer and more targeted questions would allow for grasping better-quality information and would be a more cost-effective solution.

Finally, the availability of statistically representative data can impact future research developments, which would inevitably focus on the different sets of occupational risks, as a consequence of the COVID-19 pandemic, but also as a result of wider transformations of the way we work due to digitalization of the workplaces.

## Appendix

See Tables

**Table 5:** Descriptive statistics for non-dummy demographic and economic variables

Variable	Mean	Std. dev	Min	Max
Age	42.531	11.920	15	88
Working hours	37.101	10.333	1	100
Working days for week	4.920	0.847	1	7
Make-ends-meet	3.803	1.218	1	6

^5^,

**Table 6:** Descriptive statistics for physical risk variables

Variable	Mean	SD	Min	Max
Vibrations	1.935	1.680	1	7
Noise	2.278	1.773	1	7
High temperature	2.029	1.555	1	7
Low temperature	1.868	1.386	1	7
Smoke	1.705	1.482	1	7
Vapours	1.511	1.190	1	7
Chemicals	1.732	1.443	1	7
Tobacco	1.458	1.114	1	7
Infective materials	1.618	1.410	1	7
Painful positions	2.930	1.967	1	7
Lifting persons	1.456	1.274	1	7
Loads	2.381	1.759	1	7
Sitting	3.513	2.198	1	7
Repetitive movements	3.975	2.266	1	7
Computers	3.577	2.429	1	7

^6^,

**Table 7:** Descriptive statistics for psychosocial risk variables

Variable	Mean	SD	Min	Max
Emotional disturbing	2.320	1.544	1	7
Costumer	4.146	2.455	1	7
Angry clients	2.593	1.791	1	7
Fit well	3.097	0.732	1	4
Time I want	2.077	1.097	1	5
High speed	3.703	2.013	1	7
Deadlines	3.739	2.047	1	7
Support. colleagues	4.081	0.990	1	5
Support. manager	3.774	1.165	1	5
Improve work organization	3.112	1.416	1	5
Have a say	2.261	1.443	1	5
Take a break	3.010	1.422	1	5
Enough time	3.923	0.992	1	5
Well done	4.169	0.900	1	5
Ideas	3.353	1.303	1	5
Useful	4.315	0.883	1	5
Expected	4.580	0.722	1	5
Fairly	4.192	0.913	1	5
Decisions	3.081	1.306	1	5
Hiding feelings	2.764	1.414	1	5
Appreciated	3.885	1.065	1	5
Trusted	4.158	0.910	1	5
Cooperation	4.366	0.774	1	5
Trusting manager	3.803	1.100	1	5
Get on well	4.449	0.729	1	5
Motivation	3.611	1.135	1	5

^7^.

## Footnotes

1 Data are available at [data collection]. 4th Edition. UK Data Service. SN: 8098, http://doi.org/10.5255/UKDA-SN-8098-4. The survey is implemented every five years since 1991.

2 The target population refers to all residents of the countries of interest aged 15 or older, in employment, according to the ILO definition. In each country a multi-stage, stratified, random sample is drawn. Sampling was carried out using individual-level, household-level and address-level registers, or through enumeration using a random-walk approach. Country-level samples are stratified by region and degree of urbanisation. In most countries, the target sample size was 1000, although to take into account the larger workforce in larger countries, the target was increased to 1200 in Poland, 1300 in Spain, 1400 in Italy, 1500 in France, 1600 in the UK and 2000 in Germany and Turkey. The 2015 survey was the first edition using computer-aided personal interviewing (CAPI) across all countries and all interviews. More details are in the technical report, available at https://www.eurofound.europa.eu/sites/default/files/ef_survey/field_ef_documents/6th_ewcs_-_technical_report.pdf

3 Moreover, in 2021, Eurofound produced a special edition of the EWCS with the aim of providing comparable and representative data on working conditions during the pandemic, when significant changes occurred, and the recovery process was critical. For the time being, these data have not yet been made available.

## References

[CR1] Eurofound: Sixth European Working Conditions Survey, 2015 [data collection]. 4th Edition. UK Data Service. SN: 8098 (2017)

[CR2] Agresti A (2010). Analysis of Ordinal Categorical Data.

[CR3] Arcagni A, Fattore M, Maggino F, Vittadini G (2021). (2021), Some critical reflections on the measurement of social sustainability and well-being in complex societies. Sustainability.

[CR4] Askitas N, Zimmermann KF (2015). Health and well-being in the great recession. Int. J. Manpow..

[CR5] Bacchini F, Baldazzi B, Di Biagio L (2020). The evolution of composite indices of wellbeing: an application to Italy. Ecol. Indicat..

[CR6] Bacchini F, Baldazzi B, De Carli R, Di Biagio L, Savioli M, Sorvillo M, Tinto A (2021). The evolution of the Italian framework to measure well-being. J. Off. Stat..

[CR7] Bakker, A. B., Demerouti, E. (2014), Job demands-resources theory. In: P. Y. Chen, C. L. Cooper (Eds.), *Wellbeing: A Complete Reference Guide, Work and Wellbeing*. Wiley-Blackwell, Chichester, Vol. 3, pp. 37–64.10.1002/9781118539415.wbwell019

[CR8] Bjørnskov C, Dreher A, Fischer JAV (2008). Cross-country determinants of life satisfaction: exploring different determinants across groups in society. Soc. Choice Welfare.

[CR9] Brill L, Christie F, Antoniadou M, Albertson K, Crowder M (2021). What is decent work?. A Review of the Literature.

[CR10] British Office for National Statistics: Measuring What matters – national statistician’s reflections on the national debate on measuring national well-being. Office for National Statistics, United Kingdom (2011).

[CR11] Bryson, A., Forth, J., Stokes, L.: Does worker wellbeing affect workplace performance? Department for Business Innovation & Skills, NISR (UK) (2014). https://assets.publishing.service.gov.uk/government/uploads/system/uploads/attachment_data/file/366637/bis-14-1120-does-worker-wellbeing-affect-workplace-performance-final.pdf

[CR12] Capecchi S, Simone R (2019). A Proposal for a model-based composite indicator: experience on perceived discrimination in Europe. Soc. Indic. Res..

[CR13] Cazes, S., Hijzen A., Saint-Martin, A.: Measuring and assessing job quality: The oecd job quality framework. Oecd Social, Employment and Migration Working Papers, No. 174. Oecd Publishing, Paris (2015).

[CR14] Centers for Disease Control and Prevention: Health-Related Quality of Life (HRQOL) - Well-being Concepts (2018), https://www.cdc.gov/hrqol/wellbeing.htm

[CR15] Daykin A, Moffatt P (2002). Analyzing ordered responses: a review of the ordered probit model. Underst. Stat..

[CR16] Demerouti E, Bakker AB, Nachreiner F, Schaufeli WB (2001). The job demands-resources model of burnout. J. Appl. Psychol..

[CR17] Diener E, Lucas R, Helliwell JF, Schimmack U (2009). Well-being for public policy.

[CR18] Dolan, P., Peasgood, T., White, M.: Review of Research on the Influence of Personal Well-Being and Application to Policy Making*.* UK Department for Environment Food & Rural Affairs (2006).

[CR19] Durand M (2015). The OECD better life initiative: How’s life? and the measurement of well-being. Rev. Income Wealth.

[CR20] EU-OSHA: Psychosocial Risks and Workers’ Health, OSHWiki (2013), https://oshwiki.eu/wiki/Psychosocial_risks_and_workers_health

[CR21] EU-OSHA: *Psychosocial risks and vulnerable groups*, OSHWiki (2017). https://oshwiki.eu/wiki/Psychosocial_risks_and_vulnerable_groups

[CR22] Greene, W.: Econometric Analysis, 6th (ed.) Prentice Hill Publishing. Upper Saddle River (2008).

[CR23] Guzi M, de Pedraza P (2015). A web survey analysis of subjective well-being. Int. J. Manpow..

[CR24] Hackman JR, Oldham GR (1976). Motivation through the design of work: Test of a theory. Organ. Behav. Hum. Decis. Process..

[CR25] Henseke G (2018). Good jobs, good pay, better health? The effects of job quality on health among older European workers. Eur. J. Health Econ..

[CR26] Howell RT, Kern ML, Lyubomirsky S (2007). Health benefits: Meta-analytically determining the impact of well-being on objective health outcomes. Health Psychol. Rev..

[CR27] Johnson DR, Creech JC (1983). Ordinal measures in multiple indicator models: A simulation study of categorization error. Am. Sociol. Rev..

[CR28] Jolliffe I (2011). Principal component analysis.

[CR29] Karasek RA (1979). Job demands, job decision latitude, and mental strain: Implications for job redesign. Adm. Sci. q..

[CR30] Litchfield P, Cooper C, Hancock C, Watt P (2016). Work and wellbeing in the 21st Century. Int. J. Environ. Res. Public Health.

[CR31] Maggino, F.: Challenges, needs and risks in defining well-being indicators. In Maggino, F. (Ed.), A Life Devoted to Quality of Life. Festschrift in Honor of Alex C. Michalos*,* pp. 209–233 (2016). Springer, Switzerland.

[CR32] Maxwell, R.: A New Way of Examining Job Satisfaction and Employee Well-Being: The Value of Employee Attributed Importance*,* European Association of Work and Organizational Psychology (EAWOP) (2015). http://www.eawop.org/ckeditor_assets/attachments/772/rosanna_l_maxwell_final_version.pdf?1482168576

[CR33] Nardo M, Saisana M, Saltelli A, Tarantola S (2005). Tools for composite indicators building. Eur. Comission, Ispra.

[CR34] Norman G (2010). Likert scales, levels of measurement and the laws of statistics. Adv. Health Sci. Educ..

[CR35] OECD (2013). OECD Guidelines of Measuring Subjective Well-Being.

[CR36] OECD and JRC (2008). Handbook on Constructing Composite Indicators. Methodology and User Guide.

[CR37] Oldham GR, Fried Y (2016). Job design research and theory: Past, present and future. Organ. Behav. Hum. Decis. Process..

[CR38] Olsson U (1979). Maximum likelihood estimation of the polychoric correlation coefficient. Psychometrika.

[CR39] Osterman P (2013). Introduction to the special issue on job quality: what does it mean and how might we think about it?. ILR Rev..

[CR40] Padrosa E, Belvis F, Benach J, Julià M (2021). Measuring precarious employment in the European Working Conditions Survey: psychometric properties and construct validity in Spain. Qual. Quant..

[CR41] Pittau MG, Zelli R, Gelman A (2010). Economic disparities and life satisfaction in European regions. Soc. Indic. Res..

[CR42] Pressman SD, Cohen S (2005). Does positive affect influence health?. Psychol. Bull..

[CR43] Steptoe A, Demakakos P, de Oliveira C, Banks J (2012). The psychological well-being, health and functioning of older people in England. The Dynamics of Ageing: Evidence from the English Longitudinal Study of Ageing 2002–10 Wave 5.

[CR44] Stiglitz, J., Sen, A., Fitoussi, J. P.: The measurement of economic performance and social progress revisited: reflections and overview. Report, https://hal-sciencespo.archives-ouvertes.fr/hal-01069384, Sciences Po, Paris (2009).

[CR45] Stoll L, Michaelson J, Seaford C (2012). Well-Being Evidence for Policy: A Review.

[CR46] United Kingdom Department of Health: The relationship between well-being and health (2014). https://assets.publishing.service.gov.uk/government/uploads/system/uploads/attachment_data/file/295474/The_relationship_between_wellbeing_and_health.pdf

[CR47] Veerbek, M.: A Guide to Modern Econometrics, Wiley, New York, 2nd edition (2004).

[CR48] WHO: Measurement of and target-setting for well-being: an initiative by the WHO Regional Office for Europe. World Health Organisation, Geneva (2012). http://www.euro.who.int/__data/assets/pdf_file/0003/180048/E96732.pdf

[CR49] World Bank: World development report 1990: poverty (1990). https://openknowledge.worldbank.org/handle/10986/5973.

